# A Case Report of *Pseudoxanthoma Elasticum* with Rare Sequence Variants in Genes Related to Inherited Retinal Diseases

**DOI:** 10.3390/diagnostics11101800

**Published:** 2021-09-29

**Authors:** Francesco Demetrio Lofaro, Dario Pasquale Mucciolo, Vittoria Murro, Laura Pavese, Daniela Quaglino, Federica Boraldi

**Affiliations:** 1 Department of Life Science, University of Modena and Reggio Emilia, 41125 Modena, Italy; francescodemetrio.lofaro@unimore.it (F.D.L.); daniela.quaglino@unimore.it (D.Q.); 2 Department of Neuroscience, Psychology, Drug Research and Child Health, University of Florence, Eye Clinic, 50139 Florence, Italy; dario.mucciolo@gmail.com (D.P.M.); vittoria.murro@unifi.it (V.M.); laurapavese4@gmail.com (L.P.)

**Keywords:** pattern dystrophy, retinal atrophy, ABCC6, ABCA4, IMPG1

## Abstract

A case of a patient with an early and severe visual impairment is described. Due to the occurrence of skin papules a suspect of pseudoxanthoma elasticum (PXE) was posed. PXE is a rare autosomal recessive disease clinically characterized by skin, cardiovascular and ocular manifestations, these last being those that most severely affect patients’ quality of life. A whole exome sequencing approach focusing on 340 genes related to the calcification process and/or to inherited retinal diseases (IRDs) was performed. Rare monoallelic sequence variants in *ABCA4, ABCC6*, *IMPG1*, *POC1B* and *RAX2* were found. The presence of calcified elastic fibers was assessed by ultrastructural analysis on a skin biopsy. Diagnosis of PXE was based on clinical, biomolecular and morphological results, although the additional involvement of several IRD genes is important to explain the unexpectedly severe ophthalmological phenotype of the patient also in prognostic and therapeutic perspectives. Data indicate that genetic screening using a wide-spectrum analysis approach is essential to assist ophthalmologists in improving patient counseling.

## 1. Introduction

*Pseudoxanthoma elasticum* (PXE; OMIM#264800) is a multisystem ectopic mineralization disorder characterized by fragmentation and calcification of elastic fibers [1,2]. Although several organs are involved, clinical manifestations occur mainly in the skin [3], eyes [4] and the vascular system [5]. Skin is characterized by papules in the flexural body areas which may be associated with skin laxity. The ophthalmological manifestations consist, initially, of peau d’orange, angioid streaks and comet lesions to which choroidal neovascularization (CNV) and possibly also dystrophy and atrophy are added over time, causing severe visual impairment in aging patients [6]. Mineralization of elastic fibers of blood vessels may lead to the onset of peripheral artery disease, *claudicatio intermittens* and stroke whose severity is highly variable [5,7].

Inheritance of PXE is on an autosomal recessive basis and is mainly due to pathogenic variants in the *ABCC6* gene [8], although in the last few years, the presence of pathogenic variants in calcification-related genes (e.g., vitamin K-dependent gamma-carboxylase (*GGCX*) or ectonucleotide pyrophosphatase/phosphodiesterase family member 1 (*ENPP1*)) has also been demonstrated [9,10,11]. Recently, the involvement of modifier genes has also been proposed [12]. For instance, pathogenic variants in *ABCA4* and in *USH2A* genes (i.e., genes responsible for inherited retinal diseases (IRDs)) were described in PXE patients [13,14] and these findings are of particular interest as IRDs are a group of genotypically and phenotypically heterogeneous diseases that are characterized by ocular manifestations similar to those observed in PXE.

We describe an interesting case report trying to associate genetic data (i.e., rare sequence variants in calcifying and IRD genes) and ocular findings obtained with color fundus photograph (CFP) and optical coherence tomography (OCT), two important fundus imaging examination techniques used to study retinal abnormalities.

## 2. Case Report

A 46-year-old male underwent dermatological and ophthalmologic examinations (i.e., visual acuity, color fundus photographs, optical coherence tomography [OCT (Cirrus Spectral Domain OCT; Carl Zeiss Meditec Inc., Dublin, CA, USA), fundus autofluorescence). Routinary laboratory tests including assay for magnesium, phosphorus, iron, zinc, copper, alkaline phosphatase, folic acid, vitamin B_12_, homocysteine, C-reactive protein, thyroid function and blood clotting factors were within reference range.

**Figure 1:**
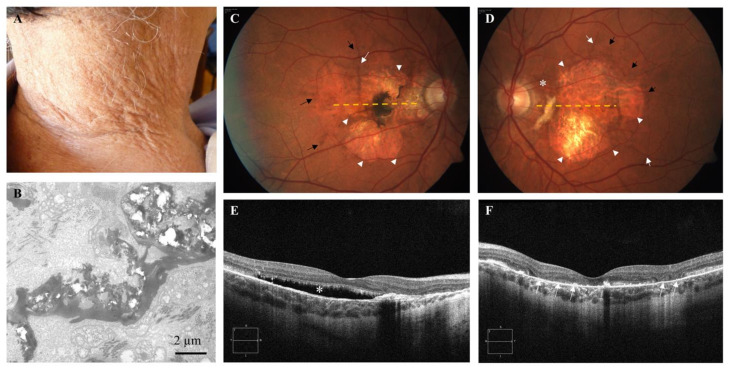
(**A**) Skin plaques in the neck area. (**B**) Mineral precipitates deforming, and cracking dermal elastic fibers are observed by transmission electron microscopy ultrastructural analysis. (**C**,**D**) Color fundus photographs of the posterior pole of the right and left eye show angioid streaks (white arrows), and macular atrophy (arrowheads) with a juxtafoveal pigmented scar in the right eye (**C**) and fibrotic tissue at the interpapillomacular region in the left eye (**D**) (asterisk). Pattern dystrophy-like changes (pigment deposition) were evident at the posterior pole (black arrows); orange dashed lines show OCT scan position. (**E**,**F**) Optical coherence tomography (OCT) examinations show subretinal fluid (asterisk) at the fovea in the right eye (**E**) and widespread RPE atrophy with atrophy of the outer retinal layers (arrows) in the left eye (**F**).

The patient was characterized by skin papules (Figure 1A) and by CNV that has been treated using photodynamic therapy (2 treatment sessions in the right eye) and intravitreal anti-vascular endothelial growth factor drugs (17 injections in the right eye and 1 injection in the left eye). Visual acuity was 6/10 in the right eye and 1/20 in the left eye. Fundus examination revealed angioid streaks and peau d’orange in addition to the presence of peripheral comet lesions and macular atrophy at the posterior pole (more severe in the left eye) with a juxtafoveal pigmented scar in the right eye and fibrotic tissue at the interpapillomacular region in the left eye. Moreover, pattern dystrophy-like changes were evident at the posterior pole. OCT scan (HD Line Raster acquisition protocol) showed subretinal fluid with the ellipsoid zone abnormalities at the fovea with juxtafoveal hyperreflective alteration corresponding to the fundus scar in the right eye. At the interpapillomacular region, the atrophy of the outer retinal layer and retinal pigment epithelium (RPE) was evident (hypertransmission phenomenon). In the left eye, OCT scan showed widespread RPE atrophy with atrophy of the outer retinal layers (Figure 1C–F).

A whole exome sequencing analysis was performed as already described [15], focusing on 340 genes known to be associated with the calcification process [11] and/or to be responsible for IRDs (RetNet, https://sph.uth.edu/retnet/; accessed on 24 May 2021).

A search of the literature or a bioinformatic analysis using the VarSome platform [16] was performed to exclude that detected rare sequence variants were benign or likely benign.

The patient was therefore characterized by monoallelic rare sequence variants on *ABCA4, ABCC6, IMPG, POC1B* and *RAX2* genes (Table 1).

As the clinical suspect of PXE was not fully supported by results of molecular analysis, a skin biopsy was performed, and ultrastructural examination was conducted to assess the presence of calcified elastic fibers [19]. Ultrastructural analysis revealed scanty collagen bundles with fibrils of heterogeneous diameter and numerous dermal elastic fibers heavily calcified (Figure 1B) and deformed by mineral deposition.

## 3. Discussion

In PXE, ocular manifestations, which can be detected in many retinal diseases, represent the most challenging disability since vision impairment adversely affects daily functions and several aspects of patients’ quality of life. Since next-generation sequencing is revolutionizing genetic testing and quickly replacing traditional methods in the diagnostic field, we adopted this approach focusing on 340 genes associated with the calcification process and/or being responsible for IRDs.

Analysis of calcification-related genes revealed only one rare sequence variant in *ABCC6*. Diagnosed PXE patients with a monoallelic *ABCC6* mutation have been already described [1], thus suggesting the involvement of other genes or the role of deep intronic variants. In our patient, a digenic inheritance as those described for *ABCC6* and *GGCX* [9] is unlikely, since all known calcification-related genes that were investigated did not show any rare sequence variant. Moreover, the occurrence of *ABCC6* pseudodominance can be excluded, since the patient’s mother, being a carrier of the same rare sequence variant, did not exhibit any sign of PXE.

The patient was characterized by pattern dystrophy-like changes, and we know that these abnormalities are very frequent in PXE-related retinopathy, especially in the advanced stages of the disease [20]. However, since the patient was still young when he developed retinal changes and ophthalmological clinical pictures (pigment deposition at the fundus, subretinal fluid and RPE abnormalities at the OCT examination) might be compatible with the evolution of a vitelliform macular lesion (vitelliform macular dystrophy pattern dystrophy subtype), the possible contribution of the *IMPG1* gene in the ocular phenotype of this patient represents an intriguing finding. *IMPG1* variants, in fact, can cause both autosomal dominant and recessive forms of vitelliform macular dystrophies [21,22], and a patient heterozygous for the c.1945C>T variant has been reported to be diagnosed with dry age-related macular degeneration (DARMD) [18]. Therefore, the detection of this variant in our patient may indicate a copresence of both pathologic conditions (i.e., PXE and DARMD) or that *IMPG1* interacts with *ABCC6* worsening the ophthalmologic PXE phenotype of the patient.

Moreover, the patient described in this report was also a carrier of a pathogenic variant in the *ABCA4* gene [17]. This gene is a member of the ABC transporter gene superfamily encoding an ATP-binding cassette transporter (retinal specific transmembrane *ABCA4* protein), which is localized to the rims of rods and cones [23]. The presence of an *ABCA4* pathogenic variant has been recently described in a patient affected by PXE, suggesting that mineralization of the Bruch’s membrane and alterations of RPE and/or of photoreceptor function can synergically contribute to the pathological phenotype [13]. The rupture of calcified Bruch’s membrane leads to choroidal neovascularization and in some cases to retinal dystrophy. However, the Bruch’s membrane calcification is not pathognomonic of PXE, since it is also present for example in age-related macular degeneration. Gliem and collaborators [24] demonstrated that PXE ophthalmological alterations and age-related macular degeneration share phenotypic similarities consistent with common pathogenic pathways. Within this context, the *ABCA4* gene is of particular interest since it is responsible for a wide range of ocular manifestations (e.g., Stargardt disease, cone–rod dystrophy, retinitis pigmentosa, age-related macular degeneration, mild fundus flavimaculatus) [25,26,27,28,29], some of which have also been described in PXE patients [1]. Although *ABCA4*-associated diseases are recessive, some patients with age-related macular degeneration or with a late-onset Stargardt’s disease were described to be carriers of a single rare *ABCA4* variant [30,31].

New rare sequence variants in *POC1B* and *RAX2* genes were found, but at present, their role in PXE remains speculative.

In the light of clinical examinations, biomolecular analyses and morphological evaluations, a diagnosis of PXE was made, and ophthalmologists were advised of the possible involvement of IRD genes in patient’s ocular manifestations, in order to explain the severity of disease progression and to improve the knowledge on ophthalmological manifestations in rare diseases.

## 4. Conclusions

Although we cannot exclude the presence of rare sequence variants and/or functional polymorphisms in other genes not yet associated with PXE and/or IRDs, which may influence the disease progression, the present report provides important information to better understand the complexity of rare diseases, since even single cases are instrumental for researchers and physicians to improve their knowledge. In particular, this study highlights that, in PXE, rare sequence variants in IRD genes can be detected, thus widening the spectrum of IRD genes that may be involved in PXE and opening the door for investigating how IRD genes can act in synergy and/or in a complementary way with the *ABCC6* gene. Therefore, genetic screening using a wide-spectrum analysis approach can help ophthalmologists to better understand eye diseases and their manifestations, improving patient counseling. In addition, genetic testing on IRD genes in PXE patients could be important also for prognostic purposes (i.e., severity and progression of ophthalmological complications) and in terms of possible gene therapies, which are in progress for several IRD subtypes and are offering, for the first time, the prospect of delaying the time course of vision loss.

## Figures and Tables

**Table 1:** List of rare sequence variants in IRD genes found in patient in heterozygous state.

Chr	Gene Symbol	Exonic Alteration	Exon	Gene Variant	Amino Acid Variant	ExAC	GnomAD	1000G	dbSNP	Reference
1	ABCA4	ns	48	c.6647C>T	p.Ala2216Val	/	/	/	/	[17]
16	ABCC6	ns	26	c.3707T>C	p.Met1236Thr	/	/	/	/	This study
6	IMPG1	ns	14	c.1945C>T	p.Leu649Phe	0.004802	0.00695	0.0014	rs118155926	[18]
12	POC1B	ns	3	c.266T>C	p.Met89Thr	0.000091	0.000032	/	rs780961965	This study
19	RAX2	ns	2	c.87G>T	p.Arg29Ser	0.000009	/	/	rs778633054	This study

Chr = chromosome; ns = nonsynonymous; ExAC = Exome Aggregation Consortium; GnomAD = Genome Aggregation Database; 1000G = 1000 Genomes; / = no data available; dbSNP = single nucleotide polymorphism database.

## Data Availability

Authors declare that all related data are available in this manuscript.
